# Rapid assembly of highly ordered DNA origami lattices at mica surfaces

**DOI:** 10.1186/s11671-025-04254-2

**Published:** 2025-05-07

**Authors:** Bhanu Kiran Pothineni, Jörg Barner, Guido Grundmeier, David Contreras, Mario Castro, Adrian Keller

**Affiliations:** 1 Technical and Macromolecular Chemistry, Paderborn University, Warburger Str. 100, 33098 Paderborn, Germany; 2 JPK BioAFM Business, Bruker Nano GmbH, Am Studio 2 d, 12489 Berlin, Germany; 3 Grupo Interdisciplinar de Sistemas Complejos and Instituto de Investigación Tecnológica, Universidad Pontificia Comillas de Madrid, 28015 Madrid, Spain

**Keywords:** DNA nanotechnology, Lattice formation, Hierarchical self-assembly, High-speed atomic force microscopy

## Abstract

The surface-assisted assembly of DNA origami lattices is a potent method for creating molecular lithography masks. Lattice quality and assembly kinetics are controlled by various environmental parameters, including the employed surface, the assembly temperature, and the ionic composition of the buffer, with optimized parameter combinations resulting in highly ordered lattices that can span surface areas of several cm^2^. Established assembly protocols, however, employ assembly times ranging from hours to days. Here, the assembly of highly ordered hexagonal DNA origami lattices at mica surfaces is observed within few minutes using high-speed atomic force microscopy (HS-AFM). A moderate increase in the DNA origami concentration enables this rapid assembly. While forming a regular lattice takes 10 min at a DNA origami concentration of 4 nM, this time is shortened to about 2 min at a concentration of 6 nM. Increasing the DNA origami concentration any further does not result in shorter assembly times, presumably because DNA origami arrival at the mica surface is diffusion-limited. Over short length scales up to 1 µm, lattice order is independent of the DNA origami concentration. However, at larger length scales of a few microns, a DNA origami concentration of 10 nM yields slightly better order than lower and higher concentrations. Therefore, 10 nM can be considered the optimum concentration for the rapid assembly of highly ordered DNA origami lattices. These results thus represent an important step toward the industrial-scale application of DNA origami-based lithography masks.

**Supplementary Information:**

The online version contains supplementary material available at 10.1186/s11671-025-04254-2.

## Introduction

Surface-assisted macromolecular self-assembly [1–6] is a promising and widely investigated strategy for the fabrication of functional surfaces and materials with promising applications in biomedicine [7], biosensing [8], and molecular electronics [9]. It is based on the adsorption of macromolecular building blocks at a solid surface under conditions that allow them to maintain some 2D mobility. These building blocks then assemble into networks and lattices via specific non-covalent interactions among themselves and/or with the surface. Various macromolecular building blocks can be used in this approach, including small organic molecules [10], peptides [11], proteins [12], and DNA [13].

DNA has proven as a particularly versatile material in surface-assisted self-assembly [14–16], as recent advances in DNA nanotechnology enable the controlled assembly of highly ordered lattices [17–19] with various symmetries and unit cells [19–22]. Such lattices are typically assembled at mica surfaces [17, 19–29] or supported lipid bilayers [30–36], but efficient lattice assembly has recently been demonstrated also at SiO_2_ surfaces [18, 37–39]. Nevertheless, mica is usually favored as a substrate because mica-assisted DNA lattice assembly it is not as sensitive to environmental parameters and thus more robust, so that lattices with an astonishing degree of order can be fabricated in a straightforward and reproducible manner [17, 18, 25, 37]. In comparison, solution-based self-assembly of DNA lattices relies solely on attractive interactions between DNA nanostructures and thus requires the precise fine-tuning of the connecting sticky or blunt ends [15, 16]. Furthermore, deposition of 2D lattices assembled in solution on solid substrates usually leads to lattice distortions and sometimes even severe lattice damage [40–42] and the resulting surface coverage is usually much lower than in the case of surface-assisted assembly, through which homogeneous lattices have been fabricated over cm^2^ surface areas [24]. The surface-assisted assembly of 2D DNA lattices thus is a robust method with various potential applications, ranging from the controlled arrangement of proteins [20, 43] and gold nanoparticles [29, 44, 45] to the fabrication of etch masks for molecular lithography patterning [38].

Despite all those advantages, some issues still need to be solved to enable the widespread application of DNA lattices assembled at solid surfaces. While the effects of several experimental parameters on lattice assembly have already been investigated and optimized to maximize lattice order [17, 23, 25] and lattice size [24], the timescales required for lattice assembly are still rather long. Depending on the monomer type and the environmental conditions, extended DNA lattices at mica and SiO_2_ surfaces are typically assembled over timespans ranging from about one hour [25] to several days [21, 22], with longer assembly times usually being favored as they result in higher lattice order [17, 23, 25] and larger surface coverage [20, 27]. For any real-world applications of DNA lattices, much shorter assembly times of the order of minutes would be highly desirable. However, previous studies already hinted at the possibility that increasing the monomer concentration may result in equivalent or even higher lattice order in a shorter time [17, 43]. Therefore, in this work, we systematically investigate the effect of monomer concentration on the assembly and quality of hexagonal DNA origami lattices at mica surfaces by high-speed atomic force microscopy (HS-AFM). We find that at a rather moderate DNA origami monomer concentration of 6 nM, densely packed DNA origami monolayers (MLs) are observed already after about 2 min, compared to 10 min at 4 nM. Intriguingly, further increases in DNA origami concentration do not result in faster ML formation. At short length scales (≤ 1 µm), no differences between DNA origami concentrations are observed once a ML has formed. This implies that high-quality DNA origami lattices can be assembled within 2 min at rather moderate DNA origami concentrations of 6 to 10 nM. However, over larger length scales of a few microns, a DNA origami concentration of 10 nM results in a slightly higher lattice order than other (higher or lower) DNA origami concentrations. We thus identified optimum conditions that enable the rapid assembly of highly ordered DNA origami lattices within a few minutes, which represents a highly important step toward the industrial-scale application of DNA-based molecular lithography masks.

## Materials and methods

### DNA origami assembly and purification

The DNA origami triangles [42] were assembled using the 7249 nt M13 mp18 scaffold (Tilibit) and 208 staples strands (Eurofins) at a tenfold staple excess in 1 × TAE (Carl Roth) containing 10 mM MgCl_2_ (Carl Roth). The solution was heated to 80 °C and subsequently cooled down to room temperature over 90 min in a thermocycler Primus 25 Advanced (PEQLAB). The folded DNA origami triangles were purified by spin filtering using Amicon Ultra 100 K filters (Millipore). The molar concentration of the purified DNA origami nanostructures was estimated by UV/Vis absorption using an Implen Nanophotometer P330.

### HS-AFM

HS-AFM was performed using a JPK Nanowizard ULTRA Speed 3 (Bruker) with USC F0.3-k0.3 cantilevers (NanoWorld) and a custom-made liquid cell. Different concentrations of DNA origami nanostructures suspended in 1 × TAE (pH 8.5) containing 10 mM CaCl_2_ (Merck) and 75 mM NaCl (VWR) were injected into the buffer-filled liquid cell to reach the final desired concentration in a total sample volume of 1 ml. The first AFM image of each experiment (indicated as time point 0 s) was recorded about 10 s after injecting the sample. Sample injection was performed manually, resulting in some variation between samples. This in particular concerns the exact timespan between injection and start of imaging, as well as the rate of injection and the place of injection within the volume of the liquid cell. Since the arrival rate of the DNA origami nanostructures at the mica surface is limited by diffusion, sample to sample variations of these parameters are responsible for differences in the number of adsorbed DNA origami triangles in the beginning of the experiments. For the first 10 min, HS-AFM images were recorded with 1 × 1 µm^2^ scan size at a line rate of 200 Hz and a resolution of 200 × 200 pixels resulting in a frame rate of 1 frame per second (fps). After about 10 min, the resolution was increased to 400 × 400 pixels, lowering the frame rate to 0.5 fps. After about another 15 min, overview images 4 × 4 µm^2^ in size were recorded at a line rate of 20 Hz and a resolution of 2048 × 2048 pixels.

### Image processing

The HS-AFM images were flattened using the batch-processing capabilities of the JPK DP Data Processing Software, except the overview images, which were processed using Gwyddion [46]. For each experiment, between 795 and 880 HS-AFM images were analyzed, i.e., 4267 images in total.

### Statistical analysis

The surface coverage *vs*. time curves were tested for statistical difference using the Compare Datasets and Fit Parameters App in Origin 2023b (OriginLab) in the dataset setting, applying the pseudo-first order model [47]. The lattice order obtained for the different concentrations after incubation for about 20 min was tested for statistical differences in Origin 2023b by One Way ANOVA using five n(Θ_60_) values from the last 50 s of each time series.

## Results and discussion

**Fig. 1:**
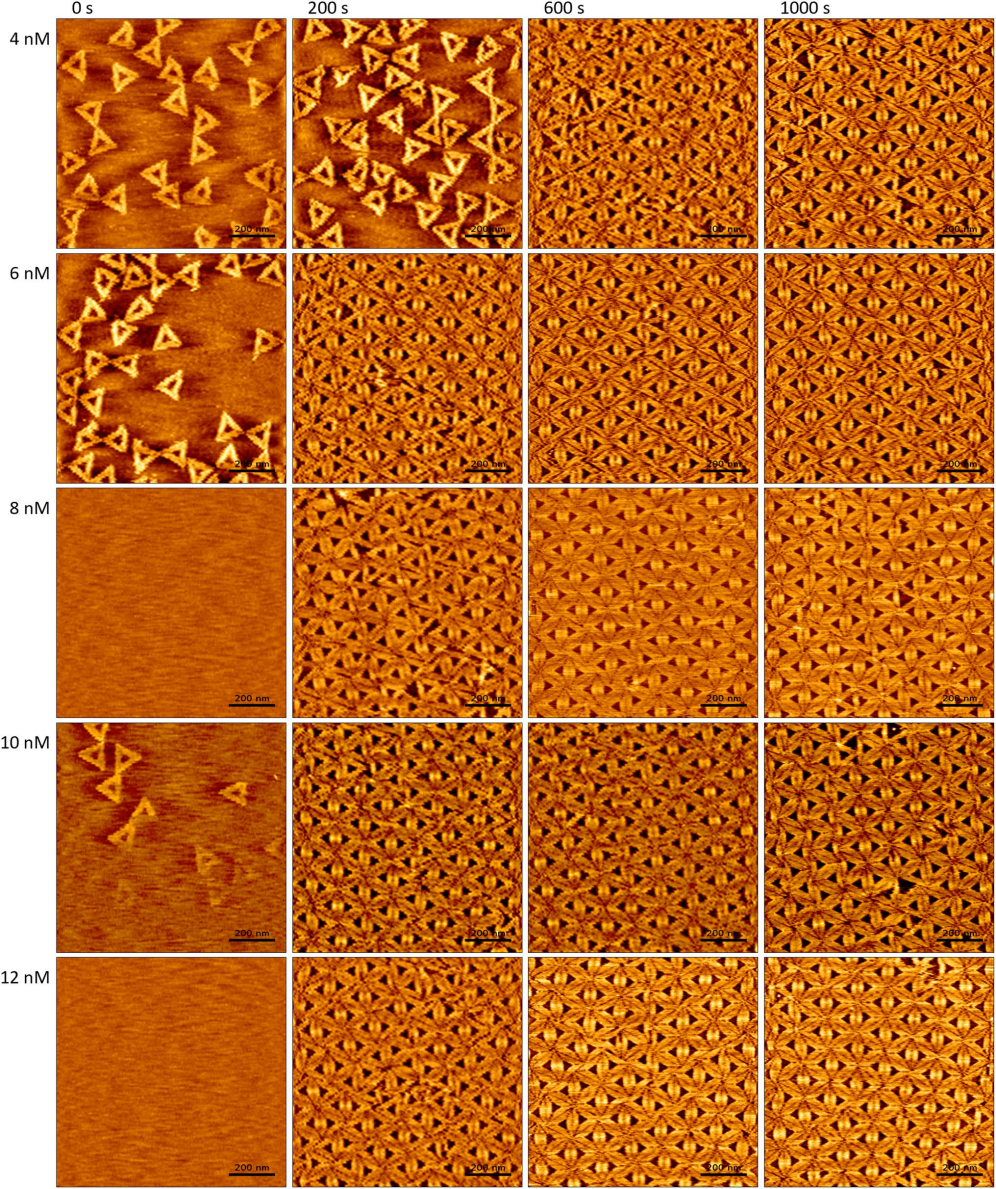
HS-AFM images (1 × 1 µm^2^) of DNA origami lattice assembly at different DNA origami concentrations recorded at different time points. The apparent differences in surface coverage at 0 s can be attributed to inhomogeneous surface coverage and variations in the manual injection of the sample solutions. The images were recorded at 1 fps. HS-AFM movies covering the whole timespan are provided in the Supplementary Material

To assess the effect of monomer concentration on DNA origami lattice assembly, we used the Rothemund triangle [42] as the monomeric building block. For these DNA origami triangles, surface-assisted lattice assembly is a result of electrostatic interactions between the charged DNA origami nanostructures and the charged mica surface, with surface coverage being maximized by arranging the adsorbed triangles in a 2D hexagonal close packed lattice. This mechanism does not require any attractive interactions between DNA origami monomers and, therefore, can proceed without the formation of nucleation seeds [25]. For this system, lattice assembly kinetics and especially lattice order can be optimized by adjusting the ionic composition of the medium, as the electrostatic interactions between the DNA origami nanostructures and the mica surface depend on the species and concentrations of available monovalent and divalent cations [17, 25]. For the current experiments, we thus selected a buffer composition that was found in our previous work to stimulate the assembly of highly ordered hexagonal lattices on mica surfaces, *i.e.*, 1 × TAE supplemented with 10 mM CaCl_2_ and 75 mM NaCl [17]. Under these conditions, a DNA origami concentration of 2 nM led to the formation of a densely packed ML in about 40 min [17]. This is verified in the HS-AFM images in Figure S1, which show only a slowly increasing surface coverage that does not yield a closed ML within 600 s of incubation. As can be seen in Fig. 1, increasing the DNA origami concentration to 4 nM leads to notably faster assembly kinetics, with a densely packed ML being formed in about 600 s. Prolonged incubation beyond 600 s leads to rearrangements within the ML, which anneals lattice defects and continuously improves lattice order [17, 25].

At a DNA origami concentration of 6 nM, lattice assembly is further accelerated, with a densely packed ML observed already after 200 s (see Fig. 1). Visual inspection does not reveal any major differences in lattice quality between the lattice obtained after 600 s at 4 nM and that obtained after 200 s at 6 nM. After prolonged incubation for 1000 s, both lattices still appear very similar. Even higher DNA origami concentrations up to 12 nM do not lead to faster lattice assembly.

We performed a topological analysis of all HS-AFM images recorded for each experiment to quantify these visual observations. To this end, our previously developed software [17, 24] was adapted to enable batch processing of large HS-AFM image stacks. The web app (available at https://github.com/mariocastro73/avator) allows to import individual images and fine tune the software parameters to obtain a 100% accurate classification of the triangles. However, as the input images differ in coverage, it is necessary to calibrate different representative parameters for different time ranges. Then, information about the location of the triangles and the coverage can be exported, so that it can be processed with another software.

**Fig. 2:**
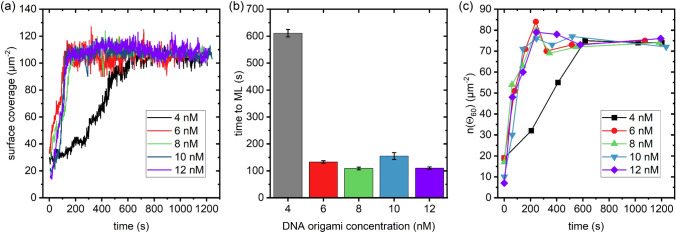
**a** Evolution of surface coverage over time. The 4 nM curve is statistically different (p < 0.001) from all other curves. **b** Time to monolayer (ML) formation extracted from the curves in a. **c** Evolution of the number of angles around 60° per µm^2^, n(Θ_60_), over time. There are no statistically significant differences in the final n(Θ_60_) values obtained at the end of the experiments

Figure 2a shows the DNA origami surface coverage as a function of incubation time for the different DNA origami concentrations. In agreement with the above qualitative assessment, surface coverage increases more slowly at a DNA origami concentration of 4 nM than at the higher concentrations, which all show a rather similar behavior. The time to ML formation identified by the surface coverage saturation is given in Fig. 2b for the different DNA origami concentrations. At a DNA origami concentration of 4 nM, ML formation occurs at about 600 s. At the higher concentrations, closed MLs are observed already after 100 to 150 s. We hypothesized that this increase in the lattice assembly rate might be related to the probability of the DNA origami triangles to form dimers in solution via blunt-end stacking at their vertices. Higher monomer concentrations may lead to increased formation of DNA origami dimers in solution, which might lead in a larger dimer-to-monomer ratio at the mica surface during the early stages of adsorption. If the adsorbed dimers acted as nucleation seeds, a larger fraction of dimers would result in accelerated lattice growth. However, a quantitative analysis of the HS-AFM images recorded at sub-ML coverage revealed no clear trend in the fraction of adsorbed dimers with monomer concentration (see Figure S2). Therefore, we rather assume that the observed decrease in the time to form a closed ML is due to an increase in the arrival rate of the DNA origami triangles at the surface, even though the limited size of the HS-AFM images allows only a very small surface area to be analyzed. The observation that a further increase in DNA origami concentration does not result in faster ML formation indicates that the rate of DNA origami nanostructures arriving at the mica surface is limited by the time it takes them to diffuse through the volume of the liquid cell. To reach a target concentration of 10 nM in these experiments, a 500 µl sample of 20 nM DNA origami solution is manually injected into the liquid cell filled with 500 µl of DNA-free buffer. To reach the surface, the DNA origami nanostructures have to diffuse along the concentration gradient. Therefore, the local DNA origami concentration at the mica-liquid interface gradually increases until it reaches the nominal target value of 10 nM, with the time to reach the target concentration being mostly independent from the monomer concentration. To test this hypothesis, we conducted a control experiment in which 28 µl of a highly concentrated DNA origami sample (360 nM) were injected into the liquid cell filled with 972 µl buffer to reach the same target concentration of 10 nM. Indeed, as can be seen in the AFM images shown in Figure S3, lattice assembly is severely delayed in this setting. Therefore, we assume that under conditions that are not limited by diffusion through the liquid cell, lattice assembly will be even faster for DNA origami concentrations of 6 nM and more.

To monitor the development of lattice order over time, we computed a Delauney triangulation (a dual geometric characterization of the Voronoi tessellation) [17, 24] and determined the distribution of triangle angles. For a perfectly packed DNA origami triangle lattice, we would expect a peaked distribution at 60°. Of course, randomness in the deposition and tiny fluctuations in the automatic triangle discovery provided by our software will broaden this distribution. However, we can expect that order is related to the height of the histogram of angles around 60° (and using ± 5° bin width). Namely, we denote this order parameter n(Θ_60_), *i.e.*, the number of angles around 60° per µm^2^. This parameter is less sensitive toward boundary effects resulting from the finite size of the AFM images than parameters based on the nearest-neighbor distribution that were used previously to quantify lattice order [17, 24].

Figure 2c shows the evolution of n(Θ_60_) over time for the different DNA origami concentrations. Interestingly, the order parameter follows the same trend as the surface coverage. It increases at early assembly times for all concentrations but saturates upon formation of a closed ML. After this point, it remains largely constant and does not exhibit any notable differences between DNA origami concentrations. This demonstrates that increasing the DNA origami concentration results in faster ML formation but does not affect the quality of the assembled lattices. Therefore, high-quality DNA origami lattices can be assembled within 2 min at rather moderate DNA origami concentrations of 6 to 10 nM.

**Fig. 3:**
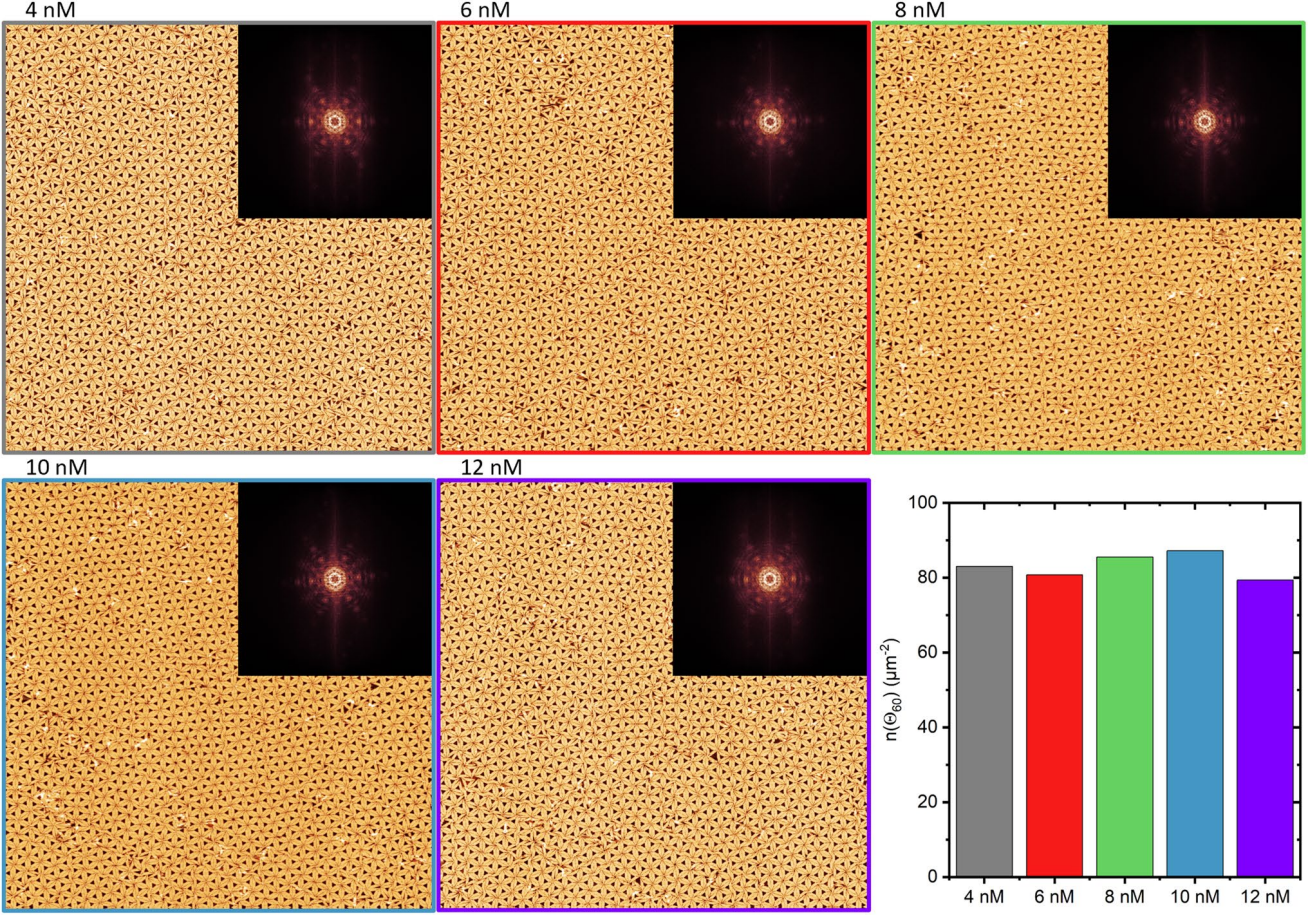
Overview AFM images (4 × 4 µm^2^) of DNA origami lattices assembled at different DNA origami concentrations recorded after approximately 25 min of incubation. The insets give the fast Fourier transforms (FFTs) of each image. The bar chart gives the number of angles around 60° per µm^2^, n(Θ_60_), calculated for the different AFM images

In the above experiments, the image size was limited by the high frame rate, so only rather small images of 1 × 1 µm^2^ could be recorded. To assess lattice quality over larger scales, overview images 4 × 4 µm^2^ in size were recorded at the end of each experiment, *i.e.*, about 25 min after sample injection. As can be seen in Fig. 3, the obtained lattices are rather similar in appearance and exhibit the same general features. In particular, the lattices are rather homogeneous over these micrometer length scales but show some point and line defects at the grain boundaries. Such defects, however, may persist even for very long times and are observed also for assembly times exceeding one hour [17, 25]. Despite the similar appearance of the different lattices, the fast Fourier transforms (FFTs) shown in the insets, reveal some differences. Even though all FFTs exhibit very pronounced correlation peaks with hexagonal symmetry, the one of the 4 nM image has a rather intense, diffuse background. With increasing DNA origami concentration, the intensity of the background decreases, until FFTs with extremely well-defined features are obtained at 8 and 10 nM concentrations. At 12 nM, however, the background reappears and the FFT again seems somewhat blurry. This is in line with the order parameter n(Θ_60_) shown in the bar chart of Fig. 3, which has slightly larger values at 8 and 10 nM than at the other concentrations. Therefore, although no differences between concentrations are observed at smaller length scales, long-range order exhibits a small maximum at an intermediate optimum DNA origami concentration of 10 nM. At larger concentrations, n(Θ_60_) is decreased again. This is because development of order requires the annealing of defects by a local rearrangement of the DNA origami lattice, which is initiated after the spontaneous desorption of single triangles from lattice sites [23, 25]. If the DNA origami concentration is too high, this rearrangement is suppressed because as soon as a lattice triangle desorbs, its site in the lattice is occupied by a new incoming triangle from the bulk solution. It should be noted, however, that Fourier and topological analyses measure different aspects of what is generally termed order [17]. Therefore, they are not fully comparable and may yield different assessments of lattice order for the same sample [17], which explains why the trend observed in the FFT images in Fig. 3 is not reproduced in all details in the n(Θ_60_) data.

In these experiments, we have monitored lattice dynamics for about 25 min. However, previous works have shown that under similar conditions, lattice order increases constantly with time even after formation of a closed monolayer, albeit at a low rate [17, 23]. Furthermore, the order parameters calculated from our in-situ HS-AFM images may be affected by the continuously scanned AFM tip. It has been demonstrated previously that high scan rates such as the one used here may notably disturb biomolecular dynamics [48] and thereby negatively affect surface-assisted biomolecular self-assembly [49]. Under static conditions without external disturbances, the formed lattices may, therefore, exhibit even higher order parameters. Therefore, we have incubated DNA origami triangles at the optimum concentration of 10 nM on mica for 20 and 67 min without continuously scanning the surface (see Figure S4). After 20 min incubation, an order parameter of n(Θ_60_) = 87 µm^−2^ is obtained, which is identical to the one observed with continuous scanning in Fig. 3. This indicates that the continuously scanned tip has only a minor influence on lattice order. However, after an additional 47 min incubation, the order parameter has increased to n(Θ_60_) = 93 µm^−2^. This verifies that longer incubation times will indeed lead to improved lattice order by the continuous annealing of defects as observed previously [17, 23]. Whether such a comparably moderate improvement in lattice order warrants longer assembly times, however, will depend on the requirements of the envisioned application.

## Conclusion

We have investigated the surface-assisted assembly of ordered DNA origami lattices at mica surfaces for different DNA origami concentrations. Using buffer conditions optimized for high lattice order and HS-AFM to monitor lattice assembly in situ at 1 frame per second, we observed the formation of ordered DNA origami lattices within minutes. At a low DNA origami concentration of 4 nM, formation of a regular lattice takes about 10 min, whereas similar lattices are obtained after 2 min for concentrations between 6 and 12 nM. Over short length scales below 1 µm, the DNA origami concentration does not affect lattice order. However, a 10 nM concentration results in slightly improved lattice order at larger scales of several microns. This concentration thus appears to be the optimum for the rapid assembly of DNA origami lattices at mica surfaces.

The observation that increasing the DNA origami concentration from 6 to 12 nM does not result in faster lattice assembly kinetics is attributed to the dominant influence of DNA origami diffusion through the bulk volume after sample injection, which limits their rate of arrival at the surface. For the routine fabrication of DNA origami lattices for molecular lithography or other applications, we thus recommend a different experimental setting, in which the substrate is getting immersed in a well-mixed solution that contains the DNA origami nanostructures already at the final target concentration of 10 nM. Under such conditions, we expect lattice assembly to occur almost instantly within less than 1 min.

## Supplementary Information

Additional file1

Additional file2

Additional file3

Additional file4

Additional file5

Additional file6

## Data Availability

Data for this article, i.e. raw HS-AFM images, are available at Zenodo at 10.5281/zenodo.14275812. The code for the analysis of the HS-AFM images can be found at https://github.com/mariocastro73/avator.
