# Supplementary material for: Rapid assembly of highly ordered DNA origami lattices at mica surfaces
Source: Discov Nano. 2025 May 7;20(1):77. doi: 10.1186/s11671-025-04254-2 (PMC12058615; doi:10.1186/s11671-025-04254-2)
Supplement: Supplementary file 1 — Additional file1 [file 11671_2025_4254_MOESM1_ESM.pdf]

Supplementary Material

Rapid assembly of highly ordered DNA origami lattices at mica surfaces

Bhanu Kiran Pothineni, Jörg Barner, Guido Grundmeier, David Contreras, Mario Castro, Adrian Keller**

HS-AFM images of DNA origami triangles at 2 nM concentration

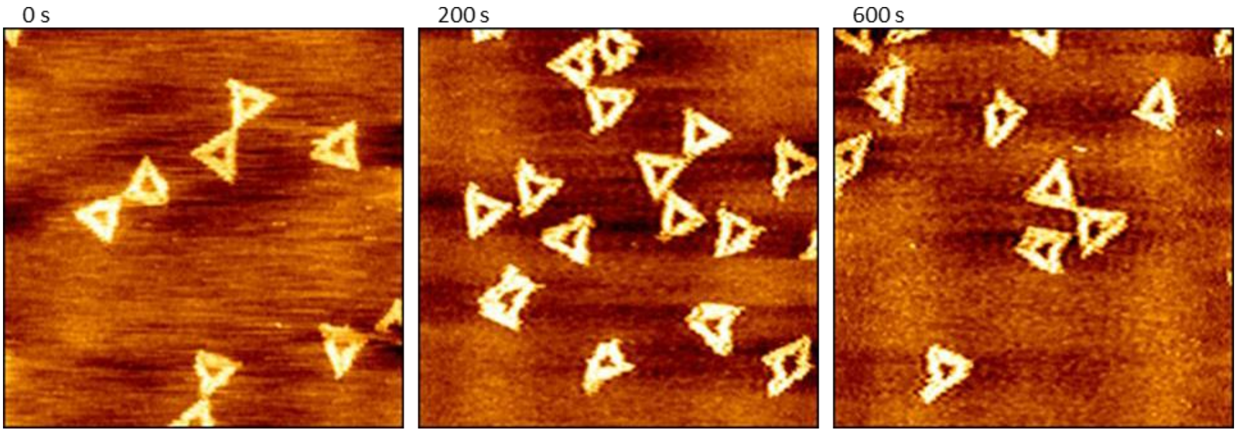

Figure S1. HS-AFM images (1 x 1 µm²) of DNA origami adsorption at a DNA origami concentration of 2 nM recorded at different time points.

Analysis of the fraction of adsorbed DNA origami dimers

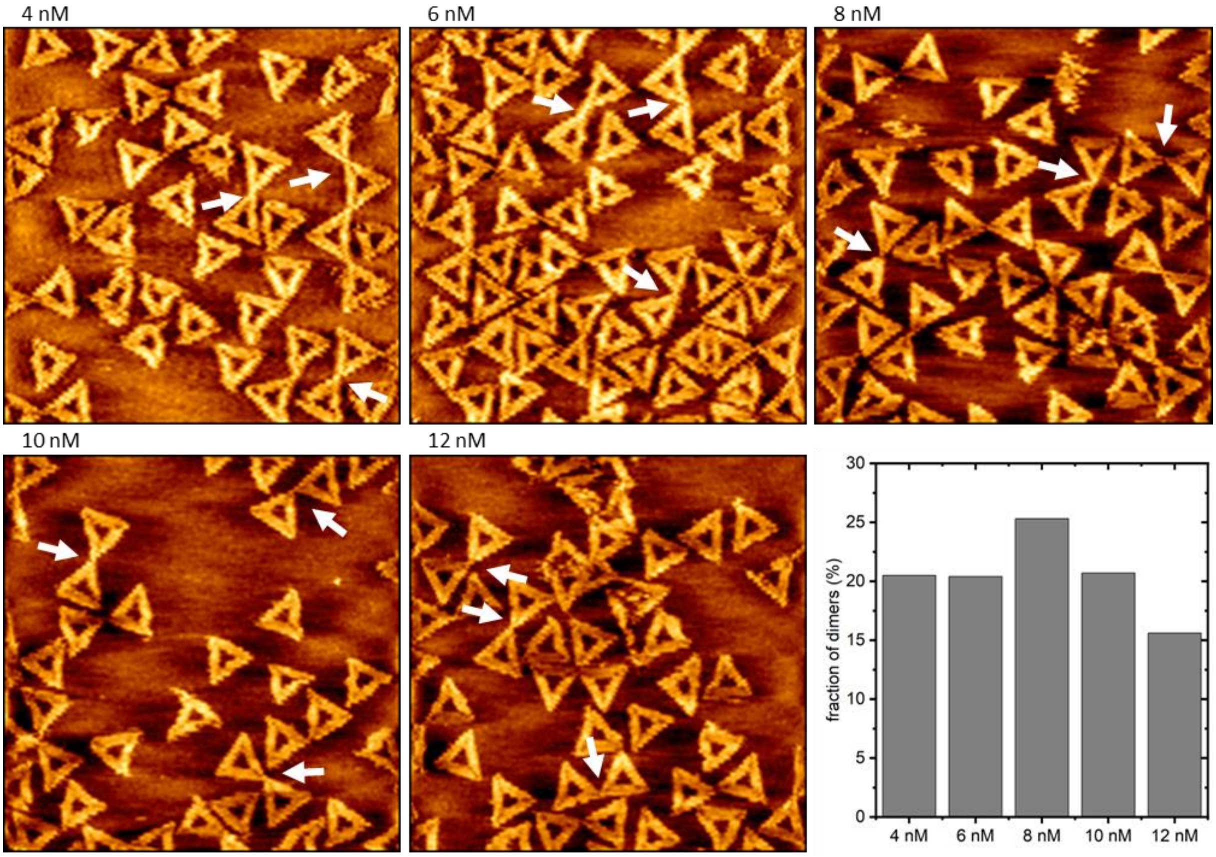

Figure S2. HS-AFM images (1 x 1 µm²) of DNA origami triangles after 200 s (4 nM), 50 s (10 nM), and 40 s (6, 8, and 12 nM), corresponding to about one third of the time to ML shown in Figure 2b. In each image, three selected DNA origami dimers are highlighted by white arrows. The bar chart gives the fraction of timers determined from the images. The few trimers and tetramers visible in some AFM images have been neglected in the analysis, as these were formed at the surface when adsorbed monomers or dimers combined.

Influence of sample injection on lattice assembly kinetics

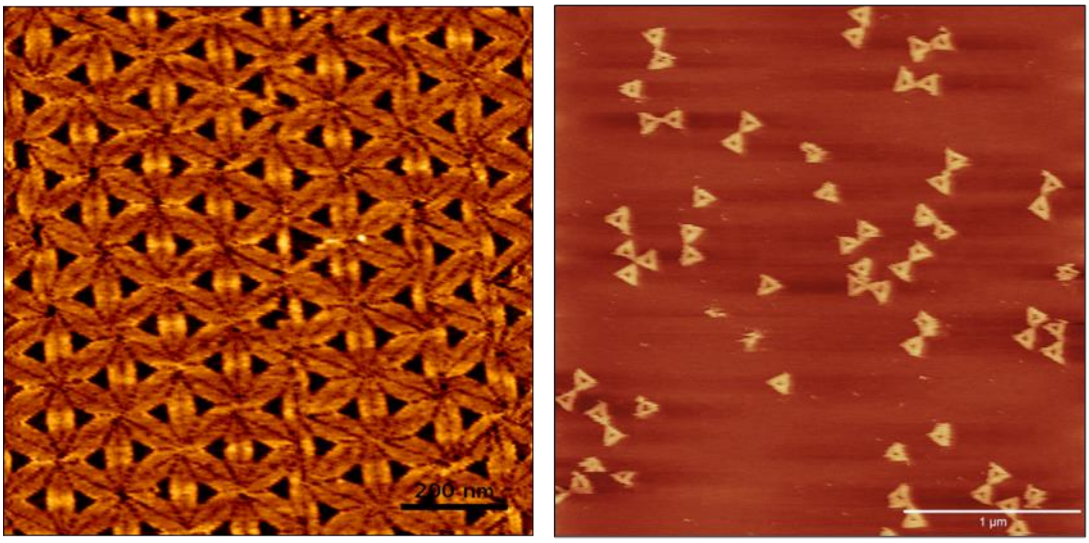

Figure S3. HS-AFM images of DNA origami triangles adsorbed on mica surfaces. Injection into the liquid cell was carried out at different sample-to-buffer volume ratios at a constant total concentration of 10 nM. Left: 500 µl sample (20 nM) injected into 500 µl DNA-free buffer. Image size 1 x 1 µm², incubation time 1242 s. Right: 28 µl sample (360 nM) into 972 µl buffer. Image size 3 x 3 µm², incubation time 2100 s.

Lattice order after long-time incubation without continuous scanning

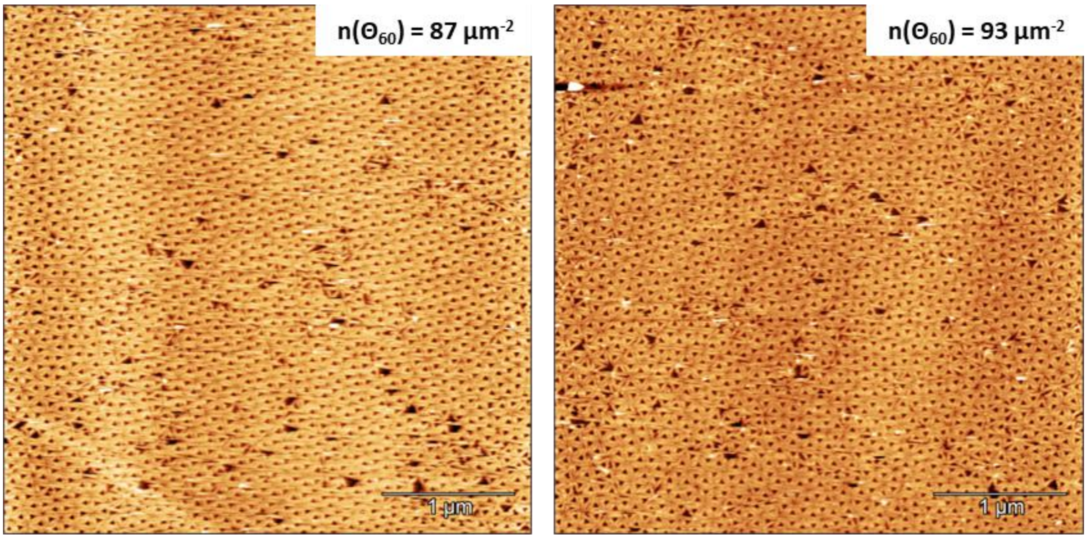

Figure S4. HS-AFM images (4 x 4 µm²) of DNA origami triangles adsorbed on surfaces after incubation for 20 min (left) and 67 min (right) without continuous scanning of the tip. The values of the order parameter $n(\Theta_{60})$ are given in the images.

Analysis of HS-AFM images of DNA origami lattices

1. Origami analysis web tool

A simple web app to analyze DNA origami triangles and lattices is freely available at https://github.com/mariocastro73/avator

Installation

Download/clone the repository and run so all the python dependencies are installed:

```
python -m pip install -r requirements.txt
```

If this does not work out of the box, create first a virtual environment and then reinstall.

```
python -m venv origami_environment
```

This will create a folder called origami_environment and, inside, you'll find the binaries to activate this environment (depending on your OS and shell).

Screenshots

Upload png (single image) or zip file (many png's)

In the case of zip files, they should be named as `save-YYYY.MM.dd-hh.mm.ss.sss.png`.

You can also export/import parameters in JSON format.

After the upload you can either *Download points (CSV)* or *Show plots*.

For single images, fine tune the parameters

Move the levers to fine tune the DNA origami detection.

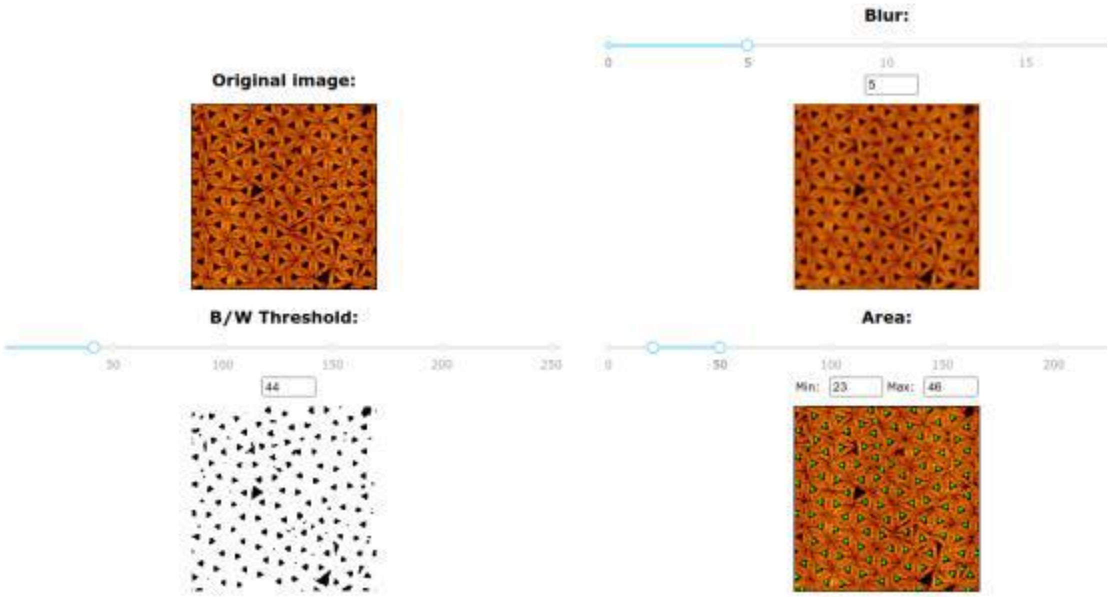

Plots for single images

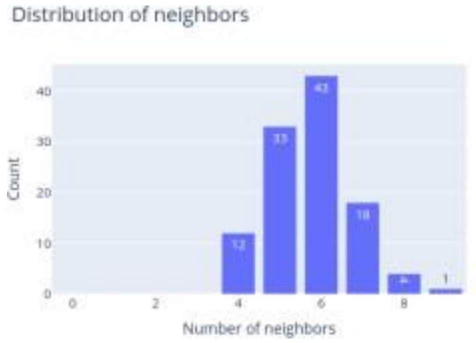

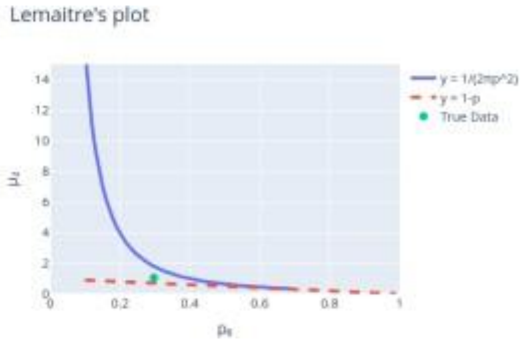

Plots for zip files

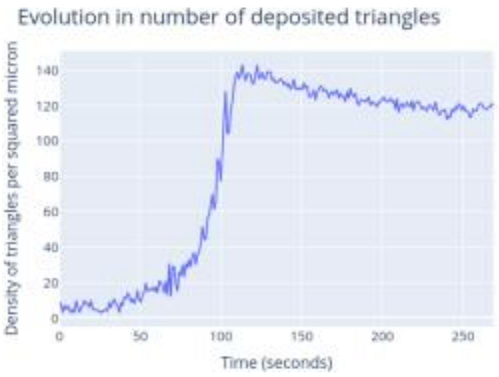

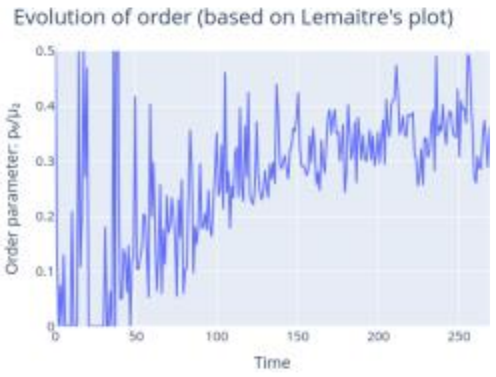

Examples in the repository

To exemplify the use of the tool, in the folder `examples` different images and their optimal configuration files can be found (see Table S1). The JSON files have a very simple structure and can also be manipulated with a simple text editor:

```
{
    "blur_level": 8,
    "bw_threshold": 41,
    "ecc_threshold": 0.85,
    "lo_area_threshold": 60,
    "hi_area_threshold": 100
}
```

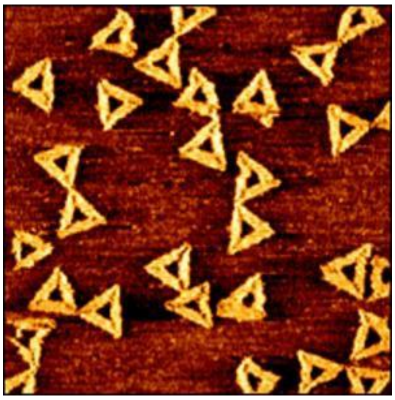	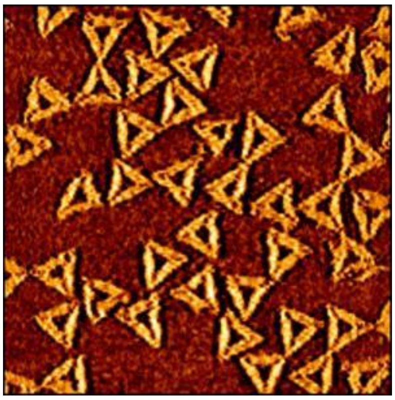	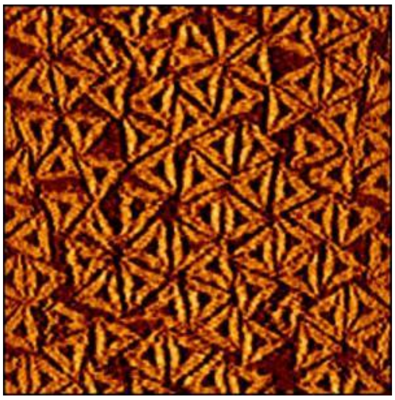
`save-2023.11.07-11.52.03.939-parameters.json`	`save-2023.11.07-11.55.29.939-parameters.json`	`save-2023.11.07-11.58.53.879-parameters.json`
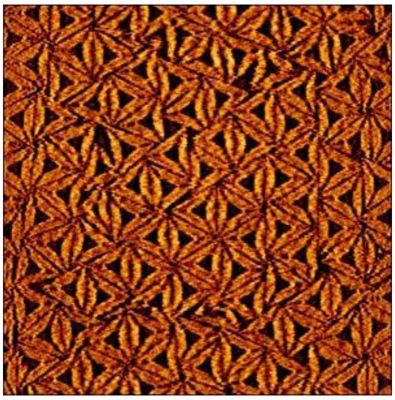	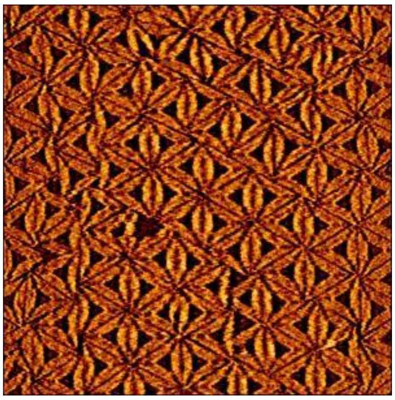	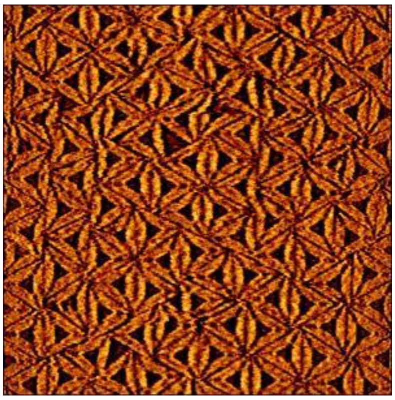
`save-2023.11.07-12.02.21.391-parameters.json`	`save-2023.11.07-12.09.05.361-parameters.json`	`save-2023.11.07-12.12.06.031-parameters.json`

Table S1. Samples in the folder `examples` with their corresponding JSON parameter file. The format of the name allows to extract the relative timestamp for bulk processing using ZIP files.

2. Additional post-processing

Function Descriptions

datavoronoi

- **Purpose**: Computes metrics related to a Voronoi tessellation for a given dataset containing x and y coordinates.Inputs:
  - `data`: A dataframe with x and y coordinates.
  - `doplot`: Logical, whether to plot the histogram of the neighbor distribution.

- **Outputs**: A list containing:
  - Lemaitre coefficients.
  - Neighbor counts and their distribution.
  - Variance of the neighbor distribution.
  - Data, triangulation, and adjacency matrix of neighbors.

- **Key Features:**
  - Calculates neighbor distributions.
  - Computes variance and other metrics from the tessellation.

---

plotlemaitre

- **Purpose**: Creates or updates a "Lemaitre's plot," visualizing relationships between Lemaitre coefficients.

- **Inputs:**
  - `output`: List generated by `datavoronoi`.
  - `add`: Logical, whether to add to an existing plot.
  - `mycex, mypch`: Parameters controlling point size and style.
  - `ymax, ymin`: Y-axis limits.
  - `defaultcol`: Color of plotted points.

- **Outputs**: Visual plot.

- **Key Features:**
  - Customizable plot based on data from Voronoi tessellation analysis.

---

generate_triangular_tessellation

- **Purpose**: Generates a grid of points forming a perfect triangular tessellation.
  - **Inputs:**
    – `n_rows, n_cols`: Number of rows and columns of triangles.
    – `spacing`: Distance between points.
- **Outputs**: Dataframe containing x and y coordinates of the tessellation.
  - **Key Features:**
    – Supports offset for alternating rows to form triangles.
    – Suitable for constructing idealized triangular grids.

---

angle_distr

- **Purpose**: Analyzes the angular distribution of triangles in a Delaunay triangulation.
  - **Inputs:**
    – `df`: Dataframe containing x and y coordinates.
- **Outputs**: Vector of angles (in degrees) for all triangles in the triangulation.
  - **Key Features:**
    – Calculates internal angles for each triangle.
    – Measures deviation from ideal equilateral triangles.

---

subtract_timestamps

- **Purpose**: Computes the time difference between two timestamp strings.
  - **Inputs:**
    – `t1, t2`: Timestamp strings in the format `HH.MM.SS.mmm`.
- **Outputs**: Time difference in seconds.
  - **Key Features:**
    – Handles conversion of timestamp strings into seconds.
    – Suitable for analyzing time intervals in data logs.

The files `main.R` and `auxiliary_functions.R` in the "R" folder contain the codes and some running examples.

The most relevant function here is `angle_distr()`. This function computes de Delauney tesselation of the points (previously exported and read into R) and their internal angles (rounding the angles to the closest "decade": 10°, 20°, ...etc. For a perfect (infinite size) ordered system, this triangular analysis would have all the angles in the 60° decade.

However, finite size effects (the triangles in the borders deviate from this) and randomness make the fraction of 60° angles much lower. However, we can use this metric as a relative measure of order.
